# Interprofessional collaboration (or lack thereof) between faculty and learning technologists in the creation of digital learning

**DOI:** 10.1186/s12909-023-04728-w

**Published:** 2023-10-04

**Authors:** Siew Ping Han, Muhammad Raihan Jumat, Jennifer Anne Cleland

**Affiliations:** 1 Lee Kong Chian School of Medicine, Nanyang Technological University, Singapore, Singapore; 2 Duke-NUS Medical School, National University of Singapore, Singapore, Singapore

**Keywords:** Digital learning, Interprofessional collaboration, Learning technologists, Educational technology, Medical education

## Abstract

**Background:**

As digital learning becomes more prevalent and important in health professions education, learning technologists play increasingly central roles in designing and delivering learning materials. However, little is understood about the process by which learning technologists have integrated into the existing teaching and learning ecosystem, and it seems that they remain marginal and undervalued. Our aim in this paper was therefore to examine the process of interprofessional co-development of course materials as experienced by educators and learning technologists.

**Methods:**

Our approach was qualitative, using individual semi-structured interviews (conducted between July 2021 to May 2022) to explore the working relationship between faculty and learning technologists. Transcripts were analysed abductively.

**Results:**

We found that the attitudes of both faculty and learning technologists towards collaborating to drive digital adoption in health professions education fell into two main themes: “embrace” and “replace” – and “conflict”, which we present as a third theme. Our results revealed that faculty did not take an active and agentic role in developing their digital practices in respect of education delivery. Learning technologists positioned themselves as a resource to support faculty’s knowledge and skill gap in digital competence. There was an obvious power differential between the two groups: learning technologists lacked agency and seemed in the position of servants to faculty masters. This created barriers to effective collaboration.

**Conclusions:**

By examining the process of co-development of course materials by faculty and learning technologists, we open up a space to examine the social, relational and organisational complexities associated with interprofessional collaboration in digital health professions education. Our study also has important implications for guiding educational policy to better position learning technologists to effectively collaborate with faculty and realise the potential of digital health professions education.

**Supplementary Information:**

The online version contains supplementary material available at 10.1186/s12909-023-04728-w.

## Background

There is increasing recognition and expectation that teaching and learning technology (TLT) should be used to improve the quality of teaching, student learning and the student experience in universities and other post-secondary education [1]. Online teaching and blended learning have now been part of teaching in higher education generally and health professions education (HPE) specifically for nearly two decades [2–5]. Advances in educational technology (EdTech) – the study, application and implementation of learning and instructional theories, practices and resources to improve learning and performance [6] – have the potential to transform higher education to deliver improved student experiences [7]. However, their actual implementation and adoption have been inconsistent, leading to high levels of variation in student learning experiences [8–10].

One reason for this variation is faculty digital competence, or the ability to access and employ digital resources for pedagogical purposes [11]. Teaching in the online environment requires different competencies from those in the face-to-face environment, particularly in the areas of technology, facilitation and engagement [12–14]. However, studies suggest that educators are not always willing to adopt digital tools [15], seeing them merely as burdensome rather than helpful [16], and may feel under-equipped to navigate digital tools due to perceived lack of technical knowledge and time to acquire the necessary skills and institutional support [17, 18].

One way in which institutions support educators is to provide technical and pedagogical support, usually from learning technologists (LT) [19, 20]. Traditionally, the role of the LT has been to “provide a bridge between the technologies and the ways in which they can be used to support learning and teaching” [21]. LTs are actively involved in managing, researching, supporting or enabling learning with the use of learning technology, combining expertise in EdTech and learning design, often playing a ‘brokerage’ role [22], which usually requires them to work closely with educators to create and deliver digital learning. This relatively new working relationship between educators and LTs is a form of interprofessional collaboration, an active partnership between colleagues with diverse backgrounds and distinctive professional cultures [23].

However, little is understood about the process by which LTs have integrated into the existing teaching and learning ecosystem. The few studies that exist on this topic suggest that while LTs play increasingly significant roles in online content creation and delivery [24], their role remains poorly defined, they are marginal institutional actors [19], and they generally have limited visibility within the teaching and learning community [25, 26]. Moreover, the role of LTs can be considered a disruption to the system. Instead of teachers having sole ownership of their modules and materials and working individually to create content, the responsibility and ownership of online learning material are shared between multiple stakeholders [27] who possess different categories of knowledge [28]. How this relationship between teachers and LT is negotiated is poorly understood and under-investigated.

Our aim in this paper was therefore to examine the process of co-development of course materials as experienced by educators and LTs. We go beyond examining faculty attitudes towards the use of EdTech to also focus on the relationship between faculty and LTs. In doing so, we “productively ask questions that explore the multifactorial circumstantial nature of introducing technology into teaching and learning activities” [29]. Our ultimate objective in doing so is to identify ways to realise the potential of technology within medical education. In doing so, we join a wider conversation within higher and health professions education, where there has been an increasing focus on the impact of digital education policies and practice on faculty, and a focus on building digital capability and developing digital practice [7, 30, 31].

## Methods

This was a qualitative study using individual semi-structured interviews for data collection. We adopted a qualitative approach given our interest was understanding rather than measuring [32]. Our study was underpinned by social constructivism, acknowledging that reality is socially constructed and context is important in the process of knowledge construction and accumulation [33].

### Study context

The study took place at one of Singapore’s three medical schools, Lee Kong Chian School of Medicine (LKCMedicine). The main mode of instruction at LKCMedicine is Team-based Learning, which takes place within an integrated eLearning ecosystem managed by a dedicated Digital Learning (DL) team comprising learning technologists, animators and programmers [34]. Online learning materials are provided to students on digital learning platforms for their self-study prior to class. The process of producing these learning materials heavily involves the DL team of LTs. Further, the study took place during the COVID-19 pandemic, when all learning activities were required to be conducted virtually, which further increased the DL team’s involvement in educational activities.

### Study background

The background to this study was a growing awareness locally that many faculty seemed under-equipped to navigate the digital tools considered necessary to develop and use online learning materials. In an earlier and separate study, we examined faculty self-efficacy using the TPACK framework [35]. TPACK comprises several knowledge domains, including domain-general and technology-specific aspects, that are relevant for teachers to implement technology in teaching and learning processes [36]. We used a previously published, self-report e-survey (Additional File 1) [37]. The survey responses indicated that our faculty tended to rate themselves more highly in the domains involving content and pedagogical knowledge, but lower in the domains involving technological knowledge (see Additional File 2). This information plus our reading of the literature informed the research question and interview questions for the current qualitative study [38, 39].

### Participants

We purposively sampled LKCMedicine core faculty and LTs from the DL team. Both groups were included so we could gain diverse perspectives on the usage of digital tools for teaching purposes and explore the relationship between faculty and DL. Unlike quantitative research, qualitative research does not predefine sample size in advance. Rather, the final number of participants is decided on the basis of the research question, those interviewed (sample specificity), the nature of the data, and the analysis approach [40]. However, as is common in studies of this nature, we anticipated 12–20 interviews would be sufficient [41]. We emailed potential interviewees from a range of levels of experience and content areas to explain the purpose of the study. Interested participants were asked to contact the main researcher directly by email and were then provided with more information about the study.

### Data collection

We designed a semi-structured interview schedule to explore (a) faculty’s experience with and attitudes towards digital tool usage, and (b) DL team members’ experience with and attitudes towards supporting faculty in digital tool usage (see Additional File 3). The interview schedule ensured consistency, but interviews were iterative so the interviewer could adapt the questions to the unique experience and role of each participant and continue until the participant felt that they had shared their experiences sufficiently. Open questions guided discussion as far as possible, supplemented by probes where required. Data were collected using the Zoom platform.

### Data analysis

Interviews were digitally audio-recorded for later transcription. Participants were anonymised during the transcription process. Data management and coding were managed manually, using Microsoft Word and Excel. Data was analysed by abductive analysis, which is “the active and interpretive process or processes researchers undertake with research evidence during a study that leads to what sense is made, meanings assigned, and from which new knowledge is presented” [42]. We initially developed a coding template based on the concepts of barriers and affordances often used to describe digital tool adoption [16] and the diffusion of innovation framework [43]. However, during the process of coding, we identified the recurring main themes of “replace”, “embrace” and “conflict” in the data and decided to shift from looking at knowledge and individual adoption, to examining attitudes and faculty-DL collaboration in more detail. Discrepancies between coders were highlighted and resolved through discussion.

### Reflexivity

Qualitative research is dependent on the relationship between the researcher and the research process [33, 44]. We considered our positions and relationships with the data continually and critically in view of our different inter-disciplinary backgrounds (SPH has a background in biomedical research and is now a medical educator and researcher. MRJ has a background in molecular virology and is now a faculty member in medical education. JAC is a clinical and occupational psychologist established in medical education and medical education research), and different levels of knowledge and experience of delivering and managing medical education, training and research. We were also cognisant of our teaching roles, attitudes towards technology, and how much we personally embraced technology in our teaching practices.

## Results

Fifteen people took part in interviews, comprising eight faculty and seven DL team members (Additional file 4). Interview lengths ranged from 15 min to one hour (median 36 min, total 543 min). Although faculty and DL were asked different sets of interview questions that were relevant to their roles in education, similar themes emerged from their responses. We identified two main themes: replace and embrace, but within each there were examples of conflict, which we present as a separate theme. The classification criteria for these three categories are described in Table 1. Henceforth, we refer to respondents demonstrating “embrace” attitudes as either faculty or DL embracers, and respondents demonstrating “replace” attitudes as either faculty or DL replacers.

Participants have been anonymised and identified as faculty (four basic scientists [coded as “B”], four clinicians [coded as “C”]) or DL. We report verbatim quotes. An ellipsis (…) indicates text that has been cut out where less relevant, and square brackets indicate any non-verbatim explanatory text. Quotations are included to aid confirmation of findings and to help the reader follow the logic of the story.

**Table 1:** Contextualisation of main themes

	Replace	Embrace	Conflict
Faculty	Low motivation to try out new digital tools, tends to stick to familiar tools Learning about digital tools is not a priority or responsibility Not aware of digital-specific affordances Top-down, forced to adopt	High motivation or interest to try out digital tools Takes personal responsibility for learning Aware of digital-specific affordances Sees pedagogical value	Has embrace attitude but unable to put it into action due to barriers
DL	Not involved in advocacy Lets faculty take the lead in requesting assistance with specific tasks Educates end-users by providing operational information	Seeks to increase awareness in end-users, has a role in advocacy Actively proposes solutions Involved in higher level/ long term planning Open to non-standard request Considers mindset change as part of educating end-users	Has embrace attitude but unable to put it into action due to barriers

### Theme 1: embrace

Faculty embracers expressed enthusiasm for digital technology in education while actively keeping up with new developments or partnering with technological specialists to tap on their expertise.

*“I feel that I, if I’m a teacher and I need to stay relevant, may need to kind of teach the current generation of students, then I need to be at least adaptable, I need to kind of ensure that I have stayed up to date with the technology and use technology as well …” (C2).*

*“… the partnering between us and the technical person is very important” (B4).*

DL embracers were pro-active in proposing digital solutions to faculty. They took on the responsibility of educating faculty about digital tools and persuading them to adopt new technologies, such as introducing them to relevant new applications, providing pedagogical training, and proposing needs-based learning solutions. For example,

*“Even before the pandemic came… [the DL department was] already looking at how to continue doing team-based learning when you cannot meet face to face. That’s why when pandemic came out, they were all prepared” (D1).*

Faculty embracers showed a deep understanding of how the current generation of students learn and interact within the online space, having “*grown up with a lot of digital tools around [them and] rely[ing] heavily on technological technology as a medium to kind of help [their] learning*” (C2). Similarly, DL embracers were knowledgeable about pedagogical principles and based their instructional design on learning outcomes. They considered how to structure content to promote maximum retention, and even questioned faculty’s approach to instructional design when appropriate. For example,

*“We will ask them whether the learning outcome is … sufficient for the material given or when we look at the material, we advise [that] actually we understand your content here, you’re talking about this … so your learning outcome should be changed to [a] certain way” (D5).*

In summary, faculty and DL embracers were enthusiastic advocates for digital tools and sought to better utilise digital affordances in teaching. Of note, DL embracers saw themselves as experts in the domain of digital pedagogy, and this was recognised by faculty embracers who valued them as partners. Even so, DL positioned themselves as advisors and faculty viewed them as such, with faculty retaining decision-making authority. Thus, while there was some interprofessional collaboration, power was unevenly distributed and largely in the hands of faculty.

### Theme 2: replace

In contrast to embracers, faculty interviewees who adopted “replace” attitudes had less knowledge about digital affordances in education and were less likely to initiate efforts to enhance digital tool adoption. They tended to see learning about digital tools as outside of the scope of their role, preferring to “*go with whatever’s easiest to use*” (B2), only adopting digital tools out of “*necessity… to deliver what [they] are asked to deliver*” (C3). Their digital tool usage was limited to direct substitutions of online tools for their existing teaching approach, for example, video recording a PowerPoint presentation rather than delivering it face-to-face.

Correspondingly, DL replacers tended to expect faculty to take the initiative in asking for support or solutions and were unlikely to proactively propose suggestions to faculty. They would assist by making standard guides or protocols available, leaving it up to faculty to contact them for more information thereafter: *“I haven’t really tried to introduce [examples of digital tools] to any [faculty]. For now we just put up in the website or Google site, that if the [faculty] see or they are interested, they will contact us” (D4).* They also tended to focus their support on *“improv[ing] how the materials are being presented, for example, adding more interactivity or in terms of aesthetic look*” (D2) without considering how these enhancements may, or may not, enhance teaching.

Thus, faculty and DL replacers had passive attitudes towards interprofessional collaboration, with both parties only engaging with each other out of necessity. They operated within separate skillsets and knowledge bases and did not explore what the other side had to offer in terms of expertise in different domains.

### Theme 3: conflict

Interestingly, those who appeared to have a predominant “embrace” attitude talked about encountering barriers which prevented them from using digital tools to their fullest potential, instead settling for a “replace” approach. Similarly, those with a predominant “replace” attitude often shared that they aspired towards doing more in terms of digital tool usage or support but faced barriers in doing so. This intrapersonal conflict between “embrace” and “replace” is explored next.

For example, while many faculty said they were open to the idea of trying new digital tools, they found teaching with unfamiliar digital tools to be challenging and complex: *“Learning [it] is like pretty difficult, I mean it is very complex… even till today I don’t, I think still there are many functionalities I don’t know” (C4).* Some struggled with the basics: *“I’m not very tech-savvy in a way, I only reach a level where I can actually read email, download…” (C1).*

Faculty considered lack of time as their biggest barrier to fully embracing digital tools. While they “*would love to have the technologies* …” (B3), more often than not “*something with a higher priority will come up*” (B2). They therefore relied heavily on the support of technical staff such as the DL team, and wanted more, or timelier, support from DL:

*“[I]f you want people to subscribe to the digital transformation, you must have enough support staff … progress is held back because you’re in the queue waiting to be supported” (B4).*

*“It’s not a hard thing to learn, but the problem is that there’s nobody to teach you” (C1).*

DL respondents conceded to this: “*It’s not because they don’t want to try, but because they really don’t have the time, they’re very busy, and so on, and the priorities are different, so they don’t have the time to invest into all these digital tool sets or learning” (D1).*

Yet, in contrast, faculty did not seem cognisant of DL’s own time constraints. DL respondents voiced frustration that faculty often acted “*like [DL had] no other work to do, but [DL could] do their work straightaway*” and approached them with “*really last minute*” requests that they were unable to support (D2). Faculty often provided inadequate support to DL in their role; for example, not providing the information DL needed to do their jobs in a timely manner: “*it’s very hard to get that information immediately*” (D3). Instead, DL had to find things out themselves and “*research about their terms and the terminology that they use… sort of like learn as a medical student also*” (D4).

Perhaps related to this, both faculty and DL recognised that certain faculty expected “*hand-holding*” (B2, C1, D2, D5) while learning about new digital tools, sometimes to the point that “*they are used to having people doing for them, that’s why they are reluctant to learn on their own*” (D7). DL also encountered challenges in convincing faculty to explore alternative digital tools, with many faculty “*prefer[ring] the traditional PowerPoint*” (D3). In addition, faculty’s lack of knowledge about technology (“*they [did] not understand how the software works*” (D1)) meant that they did not always fully appreciate DL’s approach to designing digital solutions. DL spoke of the struggle to “*get buy in*” (D1) and managing resistance *(“certain doctors, you know, say ‘I just want it to be done this way”* (D5)).

Both faculty and DL appeared to expect that the responsibility for bridging the gap in faculty’s technical knowledge fell primarily on DL’s shoulders - but DL felt disempowered to drive change forward. Instead, DL mostly saw themselves as being in an advisory or service role wherein they offered advice or took instructions from faculty, with faculty having the final decision-making authority. This mismatch between the distribution of power and responsibility led to resentment on both sides, with faculty failing to recognise the challenges faced by DL in meeting deadlines and providing technical assistance, and DL feeling overwhelmed by unrealistic demands and unappreciated for the work that they did.

## Discussion

### Main findings

Our aim in this paper was to examine the process of co-development of course materials by educators and LTs. Faculty mostly lacked pedagogical digital competence and were aware of this. While some were more open to embracing digital tools than others, overall, there was a sense that faculty did not take an active and agentic role in developing their digital practices in respect of education delivery. Instead, they saw providing content as their core (and sometimes only) role and delegated the responsibility for effective digital delivery to LTs. Correspondingly, LTs positioned themselves as a resource to support faculty’s knowledge and skill gap in digital competence. Although some LTs were more pro-active in proposing pedagogical strategies and initiating digital teaching advancements than others, generally they did not assume or expect to be equal partners in a co-development process. Instead, decision-making and delivery timelines were driven by faculty, and LTs had to work around faculty’s availability and skill level.

While we found varying degrees of interprofessional collaboration between faculty and LTs, there was an obvious power differential between the two groups. LTs lacked agency and seemed to be in the position of servants to faculty masters, wherein faculty made demands of LTs with little consideration of LTs’ workload or time constraints. In addition, LTs had limited input into the design of digital solutions even though they had more training and knowledge in digital pedagogy. As per earlier studies, it seems that LTs remain a “historically marginalized constituency” with ambiguous and undervalued roles [26].

### Comparison with previous literature

Despite the apparent advantages of embracing digital learning technology, there is much inertia in higher education institutions in doing so [45]. New technology is often adapted to existing traditional teaching practices, with lack of teachers’ digital competence, extra workload and the complexity of technological solutions presenting major barriers to innovation in teaching [46]. Similarly, our work found that faculty cited lack of technical skills and time, as well as the difficulty of mastering new digital tools, as the main barriers to embracing such technology. In addition, faculty adoption of digital tools tended to be limited to mostly substitution (direct tool substitution without functional change) and occasionally augmentation (direct tool substitution with functional improvement), the first two tiers of the SAMR model [39], which suggests technology is being used in a limited way to enhance existing teaching practices rather than extending or transforming teaching practices.

We tentatively propose that the faculty-LT relationship may parallel the relationship between doctors and other healthcare professions. Interprofessional collaboration in healthcare requires the bridging of gaps and negotiation of overlaps to overcome professional power struggles [47]. Historically, doctors have a dominant position in healthcare systems [48] and may view the expansion of others’ roles as threatening, taking actions to enforce the traditional authority gradient, even where this is counterproductive to efficient healthcare delivery [49]. Similarly, the expansion of LT roles from technical support to pedagogical design [25] has created a new professional boundary where LTs are trying to expand their practice domains while faculty try and protect their own existing position of privilege and dominance [50].

### Implications for practice and research

Rather than solely focusing on building faculty’s digital competence, it may be more fruitful to promote effective collaboration between faculty and LTs. To move beyond contesting the distribution of ownership and decisional authority over learning material [51], both parties need to adopt boundary-mitigating behaviours (consulting, mobilizing and adapting) rather than the boundary-magnifying behaviours hinted at in some of our data (enforcing, avoiding, limiting and expecting). Negotiating new boundary spaces requires relational agency, which is “the capacity for working with others to strengthen purposeful responses to complex problems” [52]. This transforms professional boundaries from barriers to relational spaces where different professionals bring their specialised practices together towards common goals. Only then can collaborators spend time doing what each knows and does best [53]. However, if poorly managed, attempts to increase LTs’ agency may be perceived as a threat to faculty dominance and generate resistance to digital adoption [54].

Effective and equal collaboration necessitates the recognition of LTs as specialists in their own right, ensuring their contribution and skills are understood and adequately valued, thus giving them more “academic capital” [55, 56] than is the case currently. We have previously proposed guidelines for building equal and synergistic partnerships between faculty and LTs [20], which include boundary-mitigating communication behaviours [51] such as:

- Consultative decision-making within learning design teams that draws upon the acknowledged expertise of both faculty and LTs.
- Mobilising behaviours to increase inclusivity and information sharing via effective feedback channels, for example through regular meetings involving both faculty and LTs.
- Adapting behaviours to build collaborative relationships, such as sharing of responsibility for and ownership of digital content between faculty and LTs.

Further, structural improvement in terms of clearer positioning of LTs within the educational ecosystem coupled with an explicit career structure may help recognise that LTs are central to supporting institutional change [19].

As per MacLeod et al. [57], we bring in the voices of a group little heard in the health professions literature, in this case, LTs. By doing so, we hope to make visible a relationship and practices which are previously unexplored, and in the background, or “back stage” [58]. This might open up these practices to a more critical eye. We suggest that future research should aim to understand the micro-mechanisms, or complex interactions, that shape the relationship between faculty and LTs, and investigate the professional cultures, power and legitimacy that underpin knowledge mobilization. Exploring the social and material assemblages required to produce technology-enhanced teaching materials would also provide a fresh gaze on this unrecognized aspect of health professions education.

It is also important to step back and think of the learners. Students have very positive attitudes towards digital technologies and see these as an important component of the learning environment [59, 60]. The lack of effective collaboration between faculty and LTs we identified has the potential to impoverish the student experience, with potential ramifications for student satisfaction and other metrics beloved by universities worldwide.

### Strengths and weaknesses

This is one study from one country with a particularly hierarchical social system which is reflected in academia [61, 62]. The relationships between faculty and LTs may be different in medical schools in different societies. This merits further comparative studies. As with any voluntary study, there would have been an element of participant self-selection. However, we took care to recruit a representative group of participants with a range of experience (e.g., junior and more senior staff) and backgrounds, and we saw the same ideas coming up over time, so we feel confident that our data reflect common experiences and views. While our number of interviews was relatively small, this is acceptable as our research question was focused, our participants “information rich”, our potential pool of participants small (the medical school has only 55 full-time faculty), and we used a lens to frame data analysis [40].

## Conclusion

By examining the process of co-development of course materials by faculty and LTs, we glimpsed the practices and relationships required to produce digital learning materials. The use of EdTech may have increased the importance of the LT, but poorly defined roles and hierarchical relationships between faculty and LTs limit the role LTs currently play in health professions education. In opening up this space, we hope this study might encourage work examining and addressing the social, relational and organisational complexities associated with digital health professions education and identify ways of realising the potential of technology in medical education.

### Electronic supplementary material

Below is the link to the electronic supplementary material.

Additional file 1

Additional file 2

Additional file 3

Additional file 4

## Data Availability

The datasets are available from the corresponding author on reasonable request.

## List of abbreviations

HPE: health professions education
LT: learning technologist
TLT: teaching and learning technology
DL: digital learning
EdTech: educational technology

## References

[1] Chen M, Lucas G (2012). Education Nation: six leading edges of innovation in our schools.

[2] Costello E, Corcoran M, Barnett J, Birkmeier M, Cohn R, Ekmekci O (2014). Information and communication technology to facilitate learning for students in the health professions: current uses, gaps and future directions. Oneline learning. Official J Online Learn Consortium.

[3] Curran V, Matthews L, Fleet L, Simmons K, Gustafson DL, Wetsch L (2017). A review of Digital, Social, and Mobile Technologies in Health Professional Education. J Contin Educ Health Prof.

[4] Ruiz JG, Mintzer MJ, Leipzig RM (2006). The impact of E-Learning in Medical Education. Acad Med.

[5] Singh V, Thurman A (2019). How many ways can we define Online Learning? A systematic literature review of definitions of Online Learning (1988–2018). Am J Distance Educ.

[6] AECT. The Definition and Terminology Committee. 2023 [Available from: https://aect.org/news_manager.php?page=17578#].

[7] Jisc. Digital learning rebooted 2020 [Available from: https://www.jisc.ac.uk/reports/digital-learning-rebooted].

[8] Bernard RM, Borokhovski E, Schmid RF, Tamim RM, Abrami PC (2014). A meta-analysis of blended learning and technology use in higher education: from the general to the applied. J Comput High Educ.

[9] Bughrara MS, Swanberg SM, Lucia VC, Schmitz K, Jung D, Wunderlich-Barillas T, Beyond (2023). COVID-19: the impact of recent pandemics on medical students and their education: a scoping review. Med Educ Online.

[10] Wu SJ, Fan YF, Sun S, Chien CY, Wu YJ (2021). Perceptions of medical students towards and effectiveness of online surgical curriculum: a systematic review. BMC Med Educ.

[11] Gudmundsdottir GB, Hatlevik OE (2017). Newly qualified teachers’ professional digital competence: implications for teacher education. Eur J Teacher Educ.

[12] Ko S, Rossen S. Teaching online: a practical guide. 4th ed. Routledge; 2017.

[13] Martin F, Budhrani K, Wang C. Examining faculty perception of their readiness to teach online. Online Learn. 2019;23(3).

[14] Wray M, Lowenthal P, Bates B, Stevens E (2008). Investigating perceptions of teaching online & f2f. Acad Exch Q.

[15] Reid P (2012). Categories for barriers to adoption of instructional technologies. Educ Inform Technol.

[16] Demirbağ M, Kılınç A (2018). Preservice teachers’ risk perceptions and willingness to use educational technologies: a belief system approach. J Educ Future.

[17] Downing JJ, Dyment JE (2013). Teacher educators’ readiness, Preparation, and perceptions of preparing Preservice Teachers in a fully online environment: an exploratory study. Teacher Educ.

[18] O’Doherty D, Dromey M, Lougheed J, Hannigan A, Last J, McGrath D (2018). Barriers and solutions to online learning in medical education - an integrative review. BMC Med Educ.

[19] Oliver M (2002). What do learning technologists do?. Innovations in Education and Teaching International.

[20] Jumat R, Loan-Ng SBL, Mogali SR, Ng KB, Leong BY, Han SP. Twelve tips for co-production of online learning. Med Teach. 2023:1–6. 10.1080/0142159X.2023.2206533 37200495

[21] Conole G, White S, Martin O, Conole G, Oliver M (2007). The impact of e-learning on organisational roles and structures. Contemporary perspectives in e-learning research.

[22] Sugrue C, Englund T, Solbrekke TD, Fossland T (2017). Trends in the practices of academic developers: trajectories of higher education?. Stud High Educ.

[23] Morgan S, Pullon S, McKinlay E (2015). Observation of interprofessional collaborative practice in primary care teams: an integrative literature review. Int J Nurs Stud.

[24] Masters K, Ellaway R (2008). e-Learning in medical education guide 32 part 2: technology, management and design. Med Teach.

[25] Ellaway R, Begg M, Dewhurst D, Macleod H (2016). In a glass darkly: Identity, agency and the role of the Learning Technologist in shaping the learning environment. E-Learning and Digital Media.

[26] Watermeyer R, Crick T, Knight C (2021). Digital disruption in the time of COVID-19: learning technologists’ accounts of institutional barriers to online learning, teaching and assessment in UK universities. Int J Acad Dev.

[27] Englander R, Holmboe E, Batalden P, Caron RM, Durham CF, Foster T (2020). Coproducing Health Professions Education: a prerequisite to Coproducing Health Care Services?. Acad Med.

[28] Çam ŞS, Erdamar Koç G. A needs analysis study on technological pedagogical content knowledge of faculty members. Educ Inform Technol. 2021.

[29] Ellaway RH. Researching technology use in health professions education: questions, theories, approaches. In: Cleland J, Durning S, editors. Researching Medical Education, Second Edition. Oxford: Wiley; 2023. p. 61–70.

[30] Englund C, Olofsson AD, Price L (2016). Teaching with technology in higher education: understanding conceptual change and development in practice. High Educ Res Dev.

[31] Jisc. Learning and teaching reimagined: Change and challenge for students, staff and leaders 2020 [Available from: https://www.jisc.ac.uk/reports/learning-and-teaching-reimagined-change-andchallenge].

[32] Cleland J. Exploring versus measuring: considering the fundamental differences between qualitative and quantitative research. In: Cleland J, Durning SJ, editors. Researching Medical Education, Second Edition. UK: Wiley; 2023. p. 3–14.

[33] McMillan W. Theory in healthcare education research: the importance of worldview. Researching Medical Education 2nd Ed. Wiley; 2023.

[34] Rajalingam P, Rotgans JI, Zary N, Ferenczi MA, Gagnon P, Low-Beer N (2018). Implementation of team-based learning on a large scale: three factors to keep in mind. Med Teach.

[35] Koehler MJ, MIshra P, Kereluik K, Shin TS, Graham CR, Spector J, Merrill M, Elen J, Bishop M (2014). The Technological Pedagogical Content Knowledge Framework. Handbook of Research on Educational Communications and Technology.

[36] Voogt J, Fisser P, Pareja Roblin N, Tondeur J, van Braak J (2013). Technological pedagogical content knowledge - a review of the literature. J Comput Assist Learn.

[37] Youm J, Corral J (2019). Technological Pedagogical Content knowledge among medical educators: what is our readiness to teach with technology?. Acad Med.

[38] O’Doherty D, Lougheed J, Hannigan A, Last J, Dromey M, O’Tuathaigh C (2019). Internet skills of medical faculty and students: is there a difference?. BMC Med Educ.

[39] Puentadura RR. SAMR and TPACK: Intro to advanced practice 2010 [Available from: http://hippasus.com/resources/sweden2010/SAMR_TPCK_IntroToAdvancedPractice].

[40] Malterud K, Siersma VD, Guassora AD (2016). Sample size in qualitative interview studies: guided by Information Power. Qual Health Res.

[41] Cleland J, Alexander K, Poobalan A. Sampling and recruiting participants. In: Rees EL, Ledger A, Walker KA, editors. Starting Research in Clinical Education. Wiley Blackwell; 2023. pp. 47–56.

[42] Earl Rinehart K (2020). Abductive Analysis in qualitative Inquiry. Qualitative Inq.

[43] Rogers EM (2003). Diffusion of innovations (5th Edition).

[44] Olmos-Vega FM, Stalmeijer RE, Varpio L, Kahlke R. A practical guide to reflexivity in qualitative research: AMEE Guide No. 149. Med Teach. 2022:1–11. 10.1080/0142159X.2022.2057287 35389310

[45] Røe Y, Wojniusz S, Bjerke AH. The digital transformation of higher education teaching: four pedagogical prescriptions to move active learning pedagogy forward. Front Educ. 2022;6.

[46] Børte K, Nesje K, Lillejord S. Barriers to student active learning in higher education. Teach High Educ. 2020:1–19.

[47] Schot E, Tummers L, Noordegraaf M (2020). Working on working together. A systematic review on how healthcare professionals contribute to interprofessional collaboration. J Interprof Care.

[48] Goldman J, Reeves S, Wu R, Silver I, MacMillan K, Kitto S (2016). A sociological exploration of the tensions related to interprofessional collaboration in acute-care discharge planning. J Interprof Care.

[49] Gilardi S, Guglielmetti C, Pravettoni G (2014). Interprofessional team dynamics and information flow management in emergency departments. J Adv Nurs.

[50] Weber CE, Kortkamp C, Maurer I, Hummers E (2022). Boundary work in response to Professionals’ contextual Constraints: micro-strategies in interprofessional collaboration. Organ Stud.

[51] Conn LG, Haas B, Cuthbertson BH, Amaral AC, Coburn N, Nathens AB (2016). Communication and culture in the Surgical Intensive Care Unit: Boundary Production and the improvement of Patient Care. Qual Health Res.

[52] Edwards A (2011). Building common knowledge at the boundaries between professional practices: relational agency and relational expertise in systems of distributed expertise. Int J Educational Res.

[53] Abramson J, Rosenthal B, Edwards L (1995). Interdisciplinary and interorganizational collaboration. Encyclopedia of social work 19th ed.

[54] Mirrlees T, Alvi S. EdTech Inc: selling, automating and globalizing higher education in the Digital Age. Routledge; 2020.

[55] Bourdieu P. Homo academicus. Stanford University Press; 1988.

[56] Watermeyer R, Chubb J (2018). Evaluating ‘impact’ in the UK’s Research Excellence Framework (REF): liminality, looseness and new modalities of scholarly distinction. Stud High Educ.

[57] MacLeod A, Kits O, Mann K, Tummons J, Wilson KW (2017). The invisible work of distributed medical education: exploring the contributions of audiovisual professionals, administrative professionals and faculty teachers. Adv Health Sci Educ Theory Pract.

[58] Goffman E (1969). The presentation of self in everyday life.

[59] Henderson M, Selwyn N, Aston R (2015). What works and why? Student perceptions of ‘useful’ digital technology in university teaching and learning. Stud High Educ.

[60] Popovici A, Mironov C (2015). Students’ perception on using eLearning Technologies. Procedia - Social and Behavioral Sciences.

[61] Hairon S, Dimmock C (2012). Singapore schools and professional learning communities: teacher professional development and school leadership in an Asian hierarchical system. Educational Rev.

[62] Tan CY, Influence of Cultural Values on Singapore School Leadership. Educational Management Administration & Leadership [Internet]. 2022;0(0). 10.1177/17411432211073414.

