# Supplementary material for: Interprofessional collaboration (or lack thereof) between faculty and learning technologists in the creation of digital learning
Source: BMC Med Educ. 2023 Oct 4;23:727. doi: 10.1186/s12909-023-04728-w (PMC10548781; doi:10.1186/s12909-023-04728-w)
Supplement: Supplementary file 1 — Additional file 1 [file 12909_2023_4728_MOESM1_ESM.pdf]

Additional file 1: Google Form survey questions

Respondents were asked to rate the following statements on a 5-point Likert scale (strongly disagree to strongly agree).

Basic Science:

I know how to solve my own technical problems.

I can learn technology easily.

I keep up with important new technologies.

I frequently play around with technology.

I know about a lot of different technologies.

I have the technical skills I need to use technology.

I have sufficient knowledge about my basic science specialty.

I can use a scientific way of thinking.

I have various ways and strategies of developing my understanding of my basic science specialty.

I know how to assess student performance in classroom teaching (e.g., preparing teaching material, lectures, practicals, team-based learning).

I can adapt my teaching based-upon what students currently understand or do not understand.

I can adapt my teaching style to different learners.

I can assess student learning in multiple ways.

I can use a wide range of teaching approaches in classroom teaching (e.g., preparing teaching material, lectures, practicals, team-based learning).

I am familiar with common student understandings and misconceptions.

I can select effective teaching approaches to guide student thinking and learning in my basic science specialty.

I know about technologies that I can use for understanding my basic science specialty.

I can choose technologies that enhance the teaching approaches for a lesson.

I can choose technologies that enhance students' learning for a lesson.

My faculty development experiences have caused me to think more deeply about how technology could influence the teaching approaches I use in classroom teaching (e.g., preparing teaching material, lectures, practicals, team-based learning).

I am thinking critically about how to use technology in classroom teaching (e.g., preparing teaching material, lectures, practicals, team-based learning).

I can adapt the use of the technologies that I am learning about to different teaching activities.

I can select technologies to use in classroom teaching (e.g., preparing teaching material, lectures, practicals, team-based learning) that enhance what I teach, how I teach and what students learn.

I can use strategies that combine content, technologies and teaching approaches that I learned about in my faculty development experiences in classroom teaching (e.g., preparing teaching material, lectures, practicals, team-based learning).

I can provide leadership in helping others to coordinate the use of basic science content, technologies and teaching approaches in my department.

I can choose technologies that enhance the content for a lesson.

I can teach lessons that appropriately combine basic science content, technologies and teaching approaches.

Clinical:

I know how to solve my own technical problems.

I can learn technology easily.

I keep up with important new technologies.

I frequently play around with technology.

I know about a lot of different technologies.

I have the technical skills I need to use technology.

I have sufficient medical knowledge about my specialty.

I can apply medical knowledge to provide optimum patient care.

I have various ways and strategies of developing my understanding of medical knowledge and patient care.

I have sufficient knowledge about practice-based learning (learning through clinical practice).

I can apply my knowledge of practice-based learning (learning through clinical practice).

I have various ways and strategies of developing my understanding of practice-based learning (learning through clinical practice).

I know how to assess student performance in classroom teaching (e.g., preparing teaching material, lectures, practicals, team-based learning).

I know how to assess student performance in a clinical setting (bedside, clinic or operating theatre).

I can adapt my teaching based-upon what students currently understand or do not understand.

I can adapt my teaching style to different learners.

I can assess student learning in multiple ways.

I can use a wide range of teaching approaches in classroom teaching (e.g., preparing teaching material, lectures, practicals, team-based learning).

I can use a wide range of teaching approaches in a clinical setting (e.g., bedside, clinic or operating theatre).

I am familiar with common student understandings and misconceptions.

I can select effective teaching approaches to guide student thinking and learning about medical knowledge and patient care in my specialty.

I can select effective teaching approaches to guide student thinking and learning about practice-based learning (learning through clinical practice).

I know about technologies that I can use for gaining medical knowledge and patient care in my medical specialty.

I know about technologies that I can use for practice-based learning (learning through clinical practice) in my medical specialty.

I can choose technologies that enhance the teaching approaches for a lesson.

I can choose technologies that enhance students' learning for a lesson.

My formal faculty development experiences have caused me to think more deeply about how technology could influence the teaching approaches I use in classroom teaching (e.g. preparing teaching material, lectures, practicals, team-based learning).

My formal faculty development experiences have caused me to think more deeply about how technology could influence the teaching approaches I use in the clinical setting (e.g., bedside, clinic or operating theatre).

I am thinking critically about how to use technology in classroom teaching (e.g., preparing teaching material, lectures, practicals, team-based learning).

I am thinking critically about how to use technology in the clinical setting (e.g., bedside, clinic or operating theatre).

I can adapt the use of the technologies that I am learning about to different teaching activities.

I can select technologies to use in classroom teaching (e.g., preparing teaching material, lectures, practicals, team-based learning) that enhance what I teach, how I teach and what students learn.

I can select technologies to use in the clinical setting (e.g., bedside, clinic or operating theatre) that enhance what I teach, how I teach and what students learn.

I can use strategies that combine content, technologies and teaching approaches that I learned about in my formal faculty development experiences in classroom teaching (e.g., preparing teaching material, lectures, practicals, team-based learning).

I can use strategies that combine content, technologies and teaching approaches that I learned about in my formal faculty development experiences in the clinical setting (e.g., bedside, clinic or operating theatre).

I can provide leadership in helping others to coordinate the use of medical content, technologies and teaching approaches in my department.

I can choose technologies that enhance the content for a lesson.

I can teach lessons that appropriately combine medical knowledge and patient care content, technologies and teaching approaches.

I can teach lessons that appropriately combine practice-based learning (learning through clinical practice) content, technologies and teaching approaches.
