# Supplementary material for: Interprofessional collaboration (or lack thereof) between faculty and learning technologists in the creation of digital learning
Source: BMC Med Educ. 2023 Oct 4;23:727. doi: 10.1186/s12909-023-04728-w (PMC10548781; doi:10.1186/s12909-023-04728-w)
Supplement: Supplementary file 2 — Additional file 2 [file 12909_2023_4728_MOESM2_ESM.pdf]

Additional file 2: TPACK survey responses

The TPACK MedEd surveys, comprising a TPACK Basic Science survey and a TPACK Clinical survey (Youm 2019), were administered online using a Google form (Online Resources 1). Survey responses were exported from Google forms into Excel for data processing. Responses were scored on a scale from 1 for strongly disagree to 5 for strongly agree and analysed using descriptive statistics. The responses from basic scientists and clinicians were aggregated by TPACK domain and the means and standard deviations were calculated (Table S1).

A total of 49 faculty members (23% response rate) participated in the survey. The Technological Knowledge (TK), Content Knowledge (CK) and Pedagogical Knowledge (PK) scores of the LKCMed population were comparable to those of other medical institutions as reported in Youm 2019 (TK = 3.4, CK = 4.5-4.7, PK = 3.9-4.4).

Table S1 Summary of TPACK MedEd Survey responses

		TK	CK	PK	PCK	TCK	TPK	TPACK
LKCMed (n=49)	Mean	3.1	4.1	3.6	3.6	3.4	3.2	3.3
	Std dev	0.7	0.8	0.7	0.8	0.8	0.6	0.8

Legend: TK - technological knowledge (know about technology); CK - content knowledge (know what to teach); PK - pedagogical knowledge (know how to teach); PCK - pedagogical content knowledge (know how to adjust teaching methods to content); TCK - technological content knowledge (know what type of technology suits content); TPK - technological pedagogical knowledge (know what type of technology suits teaching method); TPACK - combination of all the above
