# Supplementary material for: Interprofessional collaboration (or lack thereof) between faculty and learning technologists in the creation of digital learning
Source: BMC Med Educ. 2023 Oct 4;23:727. doi: 10.1186/s12909-023-04728-w (PMC10548781; doi:10.1186/s12909-023-04728-w)
Supplement: Supplementary file 3 — Additional file 3 [file 12909_2023_4728_MOESM3_ESM.pdf]

Additional file 3: Semi-structured interview questions

Faculty:

1. How long have you been using digital tools in your teaching?
2. What types of digital tools have you been using?
3. What is your motivation for using digital tools in your teaching?
4. What are your considerations when selecting specific digital tools?
5. Which of the following best describes your level of digital tool usage?

    i. Substitution: Direct tool substitute with no functional change eg. voiceover-PPT

    ii. Augmentation: Direct tool substitute with functional improvement eg. voiceover-PPT with interspersed quizzes

    iii. Modification: Significant task redesign that is supported by digital technology eg. use of simulations, interactive teaching interfaces

    iv. Redefinition: Creation of new tasks that would not be possible without digital technology eg. 3D augmented reality, personalised lessons matched to student proficiency

6. How has using digital tools helped and/or hindered your teaching?
7. What barriers do you face in digital tool usage?
8. What would motivate you to increase digital tool usage, in terms of frequency and variety?
9. Anything else you'd like to share about your digital tool usage experience?

DL:

1. What are the key roles of DL in medical education?

   Follow up: How do you think DL contributes:

   i. To the development/ improvement of learning material?
   ii. To the running of online lessons?
   iii. To making digital tools accessible?

2. What is the level of digital tool proficiency amongst faculty?
3. How do you work with faculty with different levels of digital tool proficiency?
4. What strategies do you use to encourage digital tool usage amongst faculty?
5. What barriers do you face in supporting digital tool usage amongst faculty?

   i. How does the organisation of DL affect this?
   ii. How does the content of Medical Education affect this?

6. What resources would help you encourage digital tool usage amongst faculty?
7. What suggestions do you have to improve:

   i. Communication with faculty?
   ii. Curriculum design?
   iii. Digital tool usage within Medical Education in general?
