# Supplementary material for: Interprofessional collaboration (or lack thereof) between faculty and learning technologists in the creation of digital learning
Source: BMC Med Educ. 2023 Oct 4;23:727. doi: 10.1186/s12909-023-04728-w (PMC10548781; doi:10.1186/s12909-023-04728-w)
Supplement: Supplementary file 4 — Additional file 4 [file 12909_2023_4728_MOESM4_ESM.pdf]

Additional file 5: Characteristics of interviewees

Table 1. Interviewees were selected to ensure maximum variability in terms of level of experience and area of expertise (not shown to maintain anonymity).

Category	Code	Gender	Position
Faculty	B1	F	Asst Prof
	B2	M	Assc Prof
	B3	M	Prof
	B4	M	Assc Prof
	C1	M	Clinical Tutor
	C2	M	Consultant
	C3	F	Senior Consultant
	C4	M	Asst Prof
DL	D1	M	Senior
	D2	F	Junior
	D3	F	Junior
	D4	F	Junior
	D5	F	Senior
	D6	F	Senior
	D7	M	Junior
